# Macrophage activation state determines the response to rhinovirus infection in a mouse model of allergic asthma

**DOI:** 10.1186/1465-9921-15-63

**Published:** 2014-06-07

**Authors:** Jun Young Hong, Yutein Chung, Jessica Steenrod, Qiang Chen, Jing Lei, Adam T Comstock, Adam M Goldsmith, J Kelley Bentley, Uma S Sajjan, Marc B Hershenson

**Affiliations:** 1 Department of Molecular and Integrative Physiology, University of Michigan Medical School, 48109 Ann Arbor, MI, USA; 2 Department of Pediatrics and Communicable Diseases, University of Michigan Medical School, 48109 Ann Arbor, MI, USA; 3 Medical Sciences Research Building II, 1150 W. Medical Center Drive, Ann Arbor, MI, USA

**Keywords:** Asthma, Exacerbation, IL-13, IL-17A, M2 polarization

## Abstract

**Background:**

The mechanisms by which viruses cause asthma exacerbations are not precisely known. Previously, we showed that, in ovalbumin (OVA)-sensitized and -challenged mice with allergic airway inflammation, rhinovirus (RV) infection increases type 2 cytokine production from alternatively-activated (M2) airway macrophages, enhancing eosinophilic inflammation and airways hyperresponsiveness. In this paper, we tested the hypothesis that IL-4 signaling determines the state of macrophage activation and pattern of RV-induced exacerbation in mice with allergic airways disease.

**Methods:**

Eight week-old wild type or IL-4 receptor knockout (IL-4R KO) mice were sensitized and challenged with OVA and inoculated with RV1B or sham HeLa cell lysate.

**Results:**

In contrast to OVA-treated wild-type mice with both neutrophilic and eosinophilic airway inflammation, OVA-treated IL-4R KO mice showed increased neutrophilic inflammation with few eosinophils in the airways. Like wild-type mice, IL-4R KO mice showed OVA-induced airway hyperreactivity which was further exacerbated by RV. There was a shift in lung cytokines from a type 2-predominant response to a type 1 response, including production of IL-12p40 and TNF-α. IL-17A was also increased. RV infection of OVA-treated IL-4R KO mice further increased neutrophilic inflammation. Bronchoalveolar macrophages showed an M1 polarization pattern and *ex vivo* RV infection increased macrophage production of TNF-α, IFN-γ and IL-12p40. Finally, lung cells from OVA-treated IL-4R KO mice showed reduced CD206+ CD301+ M2 macrophages, decreased IL-13 and increased TNF-α and IL-17A production by F4/80+, CD11b+ macrophages.

**Conclusions:**

OVA-treated IL-4R KO mice show neutrophilic airway inflammation constituting a model of allergic, type 1 cytokine-driven neutrophilic asthma. In the absence of IL-4/IL-13 signaling, RV infection of OVA-treated mice increased type 1 cytokine and IL-17A production from conventionally-activated macrophages, augmenting neutrophilic rather than eosinophilic inflammation. In mice with allergic airways inflammation, IL-4R signaling determines macrophage activation state and the response to subsequent RV infection.

## Background

Macrophages are innate immune cells that play a critical role in early phases of host defense against pathogens, coordination of the adaptive immune response, and the regulation of inflammation and tissue repair. Through activation signals by various ligands and environmental cues, macrophages may change their polarization state, leading to altered immune responses. In the presence of type 1 cytokines such as interferon-γ and IL-12, macrophages are activated in a classical manner (M1-polarized) and produce pro-inflammatory cytokines and toxic mediators. In the presence of the type 2 cytokines IL-4 and IL-13, alternatively-activated (M2-polarized) macrophages express a distinct pattern of phagocytic receptors [1] and produce type 2 cytokines which play a role in anti-parasitic and allergic responses, including asthma. IL-13-overexpressing transgenic mice infected with *C. neoformans* demonstrate alternatively-activated macrophages expressing Arg-1, macrophage mannose receptor (CD206) and Ym-1, as well as lung eosinophilia, goblet cell metaplasia, elevated mucus production and airway hyperreactivity [2]. Adoptive transfer of IL-4Rα+ alternatively-activated macrophages enhances eosinophilic inflammation in ovalbumin (OVA)-sensitized and -challenged mice [3]. Moreover, CD206 is increased in the asthmatic patients and correlates with the severity of asthma [4].

Viral-induced exacerbations are a major cause of morbidity in asthma. Rhinovirus (RV), a single-stranded RNA virus belonging to *Picornaviru*s family, is consistently the most frequent pathogen identified. However, the precise mechanisms underlying RV-induced asthma exacerbations are not known. Compared to normal subjects, asthmatic patients with RV infection show enhanced neutrophilic and eosinophilic inflammation in the lower airways [5], consistent with the notion that asthmatics have a qualitatively different immune response to RV infection than controls. To test this in an animal model, we exposed OVA-sensitized and -challenged mice with allergic airways disease to RV1B, a minor group virus which infects mouse epithelial cells [6]. We found that RV increased airway hyperresponsiveness and eosinophilic inflammation, and that RV colocalized with eotaxin-producing, CD68+ lung macrophages *in vivo.* Macrophages from OVA-treated mice showed increased expression of arginase-1, Ym-1 and Mgl-2, indicating a shift in macrophage activation status, and RV inoculation of lung macrophages from OVA-treated mice induced expression of eotaxin-1, IL-4, and IL-13 *ex vivo*. Depletion of macrophages from OVA-sensitized and -challenged mice reduced eosinophilic inflammation following RV infection. Together, these results suggest that RV causes asthma exacerbations in part by infection of alternatively-activated macrophages. Finally, exposure of macrophages from naïve mice to IL-4 and IL-13 significantly increased RV-induced eotaxin mRNA expression, consistent with the notion that type 2 cytokines are sufficient to alter the response of macrophages to RV infection.

In the present study, we hypothesized that IL-4 signaling regulates the state of macrophage activation and the pattern of RV-induced exacerbation in mice with allergic airways disease. We anticipated that, in the absence of IL-4/IL-13 signaling, RV infection would preferentially increase type 1 cytokine production from conventionally-activated macrophages, augmenting neutrophilic rather than eosinophilic inflammation. To test this, we sensitized and challenged wild-type and IL-4 receptor knockout (IL-4R KO) mice with OVA and then infected these animals with RV. We found that OVA-treated IL-4R KO mice showed neutrophilic airway inflammation which was exacerbated by RV infection. Macrophages from OVA-treated mice showed an M1 polarization pattern and expressed type 1 cytokines and IL-17A in response to *ex vivo* RV infection. OVA-treated IL-4R KO mice showed decreased IL-13 and increased TNF-α and IL-17A production with RV infection by F4/80+, CD11b+ macrophages, demonstrating that IL-4R signaling determines macrophage activation state and the response to subsequent RV infection.

## Methods

### Generation of HRV

HRV1B (ATCC, Manassas, VA) were grown in cultured HeLa cells, concentrated, partially purified and titered as described previously [7]. Similarly concentrated and purified HeLa cell lysates were used for sham infection.

### OVA sensitization and challenge

All animal experiments were approved by the University of Michigan Institutional Animal Care and Use Committee. Female 8 wk-old BALB/c mice (Jackson Laboratories, Bar Harbor, MA) or age-matched BALB/c-*Il4ra*^*tm1Sz*^/J IL-4R KO mice (Jackson Laboratories) were injected intraperitoneally on days 0 and 7 with 0.2 ml PBS or a solution of alum and 100 μg endotoxin-free OVA (Sigma-Aldrich, St. Louis, MO). Next, mice were challenged intranasally with 50 μl of PBS or 100 μg OVA on days 12 and 13.

### RV exposure

Selected mice were inoculated intranasally with 50 μl of 1 × 10^8^ TCID_50_/ml RV1B, or an equal volume of sham control on day 14, 24 hours following the last OVA treatment. Lungs were collected 24 hr post infection for further analysis.

### Assessment of airway responsiveness

Airway cholinergic responsiveness was assessed by measuring changes in total respiratory system resistance in response to increasing doses of nebulized methacholine, as described previously [8]. Mice were anesthetized with sodium pentobarbital (50 mg/kg mouse, intraperitoneal injection) and a tracheostomy performed. Mechanical ventilation was conducted and total respiratory system measured using a Buxco FinePointe operating system (Buxco, Wilmington, NC). Airway responsiveness was assessed by measuring changes in resistance in response to increasing doses of nebulized methacholine.

### Bone marrow-derived macrophage cultures

Femurs were harvested from naïve wild-type BALB/c and IL-4R KO mice and the bone marrow was used to expand macrophages for *in vitro* analyses. Dissociated marrow cells were plated onto 12-well culture dishes at 1 × 10^6^ cells/ml and cultured for 6 days in RPMI supplemented with 30% L929-conditioned medium and 10% heat-inactivated fetal bovine serum [9]. Cells were stimulated for 1.5 h with medium or RV1B (multiplicity of infection, 10) and RNA harvested 8 h after infection.

### Mouse bronchoalveolar inflammatory cells and macrophage culture

Bronchoalveolar lavage (BAL) was performed using 1 ml PBS aliquots, and differential cell counts were performed using hematoxylin and eosin. BAL fluid from PBS- and OVA-treated mice was seeded in 24 well plates. BAL macrophages were purified by plastic adherence, which yielded >90% purity. Cells were stimulated for 1.5 h in the presence or absence of HRV1B (multiplicity of infection, 10), and harvested 8 or 24 h after infection for RNA and protein analysis.

### Cytokine/chemokine expression

Lung RNA was extracted with Trizol (Sigma-Aldrich) and analyzed for cytokine and chemokine gene expression by quantitative real-time PCR using specific primers and probes. Signals were normalized to GAPDH. Primer sequences are shown in the Table 1. BAL fluid was spun for 15 min at 1500 rpm, and the supernatants were analyzed for cytokine protein by multiplex immune assay (Bio-Rad, Hercules, CA) or ELISA (R&D Systems, Minneapolis, MN).

**Table 1:** Primer sequence for real-time quantitative PCR

**Gene**	**Forward primer (5′ to 3′)**	**Reverse primer (5′ to 3′)**
*Gapdh*	GTC GGT GTG AAC GGA TTT G	GTC GTT GAT GGC AAC AAT CTC
*Ifng*	TGG CTG TTT CTG GCT GTT AC	TCC ACA TCT ATG CCA CTT GAG TT
*Il12p40*	CTC CTG GTT TGC CAT CGT TT	GGG AGT CCA GTC CAC CTC TA
*Il13*	CCT GGC TCT TGC TTG CGT	GGT CTT GTG TGA TGT TGC TCA
*Il4*	GGT CTC AAC CCC CAG CTA GT	GCC GAT GAT CTC TCT CAA GTG AT
*Tnfa*	ATG CAC CAC CAT CAA GGA CTC AA	ACC ACT CTC CCT TTG CAG AAC TC
*Il1b*	TGG CAG CTA CCT GTG TCT TT C	GGA TGG GCT CTT CTT CAA AGA TG
*Cxcl1*	TGC ACC CAA ACC GAA GAA GTC AT	CAA GGG AGC TTC AGG GTC AAG
*Cxcl2*	GCG CTG TCA ATG CCT GAA G	CGT CAC ACT CAA GCT CTG GAT
*Il17a*	CCA CGT CAC CCT GGA CTC	CGG CAC TGA GCT TCC CAG AT
*Il6*	GGA GAG GAG ACT TCA CAG AGG ATA C	TCC ACG ATT TCC CAG AGA ACA
*Arg1*	AAG AAT GGA AGA GTC AGT GTGG	GGG AGT GTT GAT GTC AGT GTG
*Ym1*	AGA AGG GAG TTT CAA ACC TGG T	GTC TTG CTC ATG TGT GTA AGT CA
*Ccl24*	ACC TCC AGA ACA TGG CGG GC	AGA TGC AAC ACG CGC AGG CT
*Cxcl13*	CTC CAG GCC ACG GTA TTC TG	CCA GGG GGC GTA ACT TGA AT

### Histology, immunohistochemistry and immunofluorescence microscopy

Lungs were fixed with 10% formaldehyde overnight and paraffin embedded. Blocks were sectioned at 500 μm intervals at a thickness of 5 μm and each section was deparaffinized, hydrated and stained with hematoxylin and eosin. Other sections were stained with rabbit anti-mouse IL-17A (Abcam, Cambridge, MA). For immunohistochemistry, sections were incubated with biotinylated secondary goat-IgG, ABC reagent (Vector Laboratories, Burlingame, CA), diaminobenzidine (DAB, Sigma-Aldrich) and Gill’s hematoxylin (Fisher Scientific, Kalamazoo, MI). For fluorescence microscopy, slides were incubated with Alexa Fluor (AF)-555-conjugated rat anti-mouse CD68 and AF488-conjugated rabbit anti-mouse IL-17A or AF-conjugated isotype control IgGs. Nuclei were stained with 4′,6-diamidino-2-phenylindole (DAPI). Images were visualized using a Olympus IX71inverted phase/epifluorescence microscope and digital CCD camera.

### Flow cytometric analysis

Lungs from mice were perfused with PBS containing EDTA, minced and digested in collagenase IV. Cells were filtered and washed with RBC lysis buffer, and stimulated with for 5 h with cell stimulation cocktail (eBioscience, San Diego, CA) containing PMA, ionomycin and protein export blockers. After stimulation, dead cells were stained with Pac-Orange Live/Dead fixable dead staining dye (Invitrogen, Carlsbad, CA). Cells were then stained with anti-CD45-PacBlue, anti-TCRβ-FITC, anti-F4/80-PE-Cy5, anti-CD11c-APC, anti-CD11b-APC-Cy7, anti-CD68-PerCP-Cy5.5, anti-CD301 conjugated with Alexa Fluor (AF)-633 and anti-CD206 conjugated with AF488 (all antibodies from Biolegend, San Diego, CA). Cells were fixed, permeabilized and incubated with anti-IL-13-PE (eBioscience), anti-IL-17A-PE-Cy7 antibody (Biolegend) or anti-TNF-α-PE-Cy7 antibody (Biolegend). Stained cells were subjected to flow cytometry and analyzed on a BD Biosciences FACSAria II (BD Biosciences, San Jose, CA). Data were collected using FACSDiva software and analyzed using FlowJo software (Tree Star, Ashland, OR). For controls, we substituted IgG for all antibodies or used an FMO (fluorescence minus one) control in which all antibodies were included except that against the intracellular antigen.

### Data analysis

Data are represented as mean ± SEM. Statistical significance was assessed by student’s t test, unpaired t test, one-way analysis of variance (ANOVA), two-way ANOVA, ANOVA based on ranks, as appropriate. Differences were pinpointed by the Bonferroni or Newman-Keuls multiple comparisons test.

## Results

**Figure 1:**
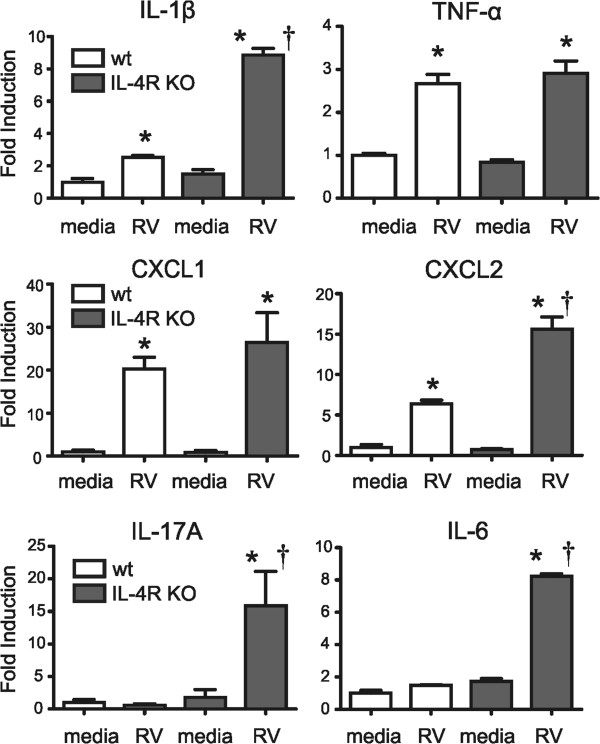
**Responses of bone marrow-derived macrophages to RV infection*.*** Dissociated marrow cells from wild-type and IL-4R KO mice were differentiated to macrophages in L929-conditioned medium. Compared to control cells, IL-4R KO cells demonstrated increased RV-induced cytokine responses. (Mean ± SEM, n = 3, *different from medium, *P* < 0.05, one-way ANOVA; †different from wild-type, *P* < 0.05, one-way ANOVA).

We previously found that, in OVA-sensitized and -challenged mice with allergic airway inflammation, RV infection increases eotaxin-1, IL-4 and IL-13 production from alternatively-activated (M2 polarized) airway macrophages, further enhancing eosinophilic inflammation and airways hyperresponsiveness [6]. We also found that *in vitro* exposure of macrophages from naïve mice to IL-4 and IL-13 significantly increased RV-induced eotaxin mRNA expression, consistent with the notion that type 2 cytokines are sufficient to alter the response of macrophages to RV infection. In the present study, we hypothesized that IL-4 signaling determines the state of macrophage activation and pattern of RV-induced exacerbation in mice with allergic airways disease. First, we tested the responses of bone marrow-derived macrophages from naïve wild-type and IL-4R KO mice. We anticipated that the loss of IL-4R signaling would increase type 1 (IL-1β, TNF-α) and IL-17A-dependent (IL-17A, CXCL1, CXCL2, IL-6) responses [10-16] to RV. Dissociated marrow cells were differentiated to macrophages in L929-conditioned medium [9]. Macrophages from naïve wild-type mice showed significant increases in IL-1β, TNF-α, CXCL1 and CXCL2 mRNA expression in response to RV infection *ex vivo* (Figure 1). Bone marrow-derived macrophages from naïve IL-4R KO cells also showed increases in IL-1β, TNF-α, CXCL1 and CXCL2 mRNA expression. However, compared to control cells, IL-4R KO cells demonstrated significantly increased IL-1β and CXCL2 in response to RV infection. Further, in contrast to cells from wild-type mice, RV infection of macrophages from naïve IL-4R KO mice significantly increased mRNA expression of IL-17A and IL-6. These results suggest that, in the absence of IL-4R signaling, macrophages show an exaggerated type 1 phenotype and increased IL-17A mRNA expression in response to RV infection.

**Figure 2:**
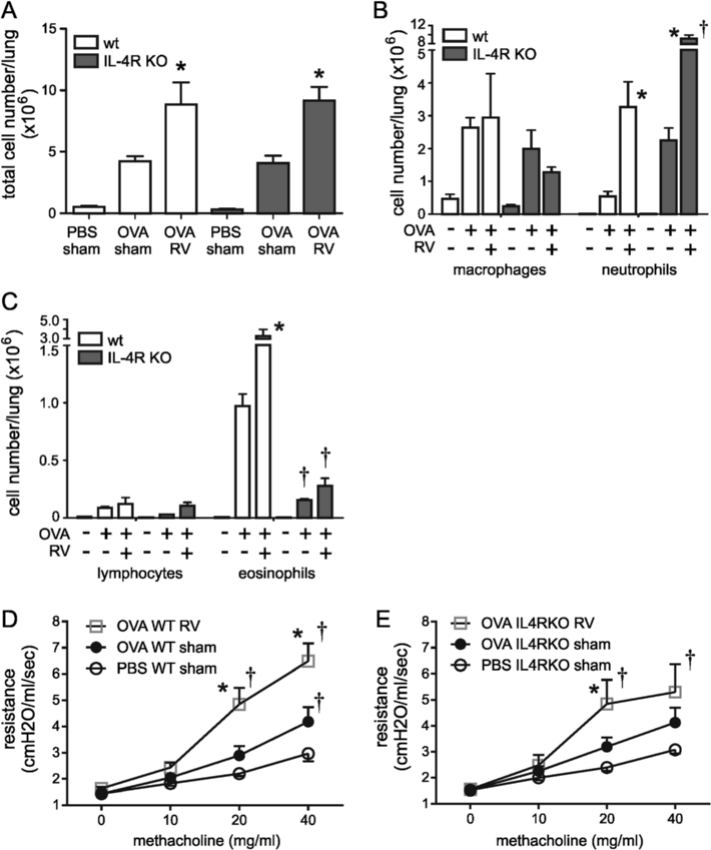
**Airway inflammation and airway hyperresponsiveness in OVA-treated wild-type and IL-4R KO mice.** Eight-week old wild-type or IL-4R KO mice were treated with PBS or OVA and inoculated with sham or RV. Bronchoalveolar lavage was performed 24 hour post-inoculation. After counting the total number of cells, cytospins were performed and stained with hematoxylin and eosin, and differential counts determined from 200 cells. The identity of neutrophils and eosinophils was confirmed by immunofluorescence staining for neutrophil elastase and major basic protein (not shown). **(A)** RV infection increases the total number of BAL cells per lung in OVA-sensitized and -challenged wild-type and IL-4R KO mice. **(B)** RV infection increases the number of airway neutrophils per lung in OVA-treated wild-type and IL-4R KO mice. The neutrophil response was significantly higher in IL-4R KO mice. **(C)** RV infection increases the number of airway eosinophils per lung in OVA-treated wild-type mice. The eosinophil response was significantly attenuated in IL-4R KO mice. (Mean ± SEM, n = 3, *different from medium, p < 0.05, one-way ANOVA; †different from wild-type, p < 0.05, one-way ANOVA.) **(D & E)** Airway cholinergic responsiveness was assessed by measuring changes in total respiratory system resistance in response to increasing doses of nebulized methacholine. Data from wild type **(D)** and IL-4R KO mice **(E)** are shown. (Mean ± SEM, n = 4-6 in each group, *different from sham, *P* < 0.05, two-way ANOVA; †different from PBS, *P* < 0.05, two-way ANOVA).

Next, we examined the effect of IL-4R KO in mice sensitized and challenged with OVA and infected with RV. As previously [17], RV alone had modest effects on airway neutrophilic inflammation and responsiveness (not shown). As expected, OVA treatment of wild-type mice significantly increased the total number of BAL cells (Figure 2A), augmenting the number of neutrophils and eosinophils (Figure 2B and C). RV infection of wild-type mice further increased lung inflammation, resulting in an additional 2-fold increase in BAL cells. Both neutrophils and eosinophils were increased in the BAL following RV infection. In IL-4R KO mice, OVA sensitization and challenge was also accompanied by a significant increase in BAL inflammatory cells (Figure 2A). The inflammatory cells consisted nearly exclusively of neutrophils, and the number of eosinophils in the airways was significantly reduced compared to wild-type mice (Figure 2B and C). When OVA-treated IL-4R KO mice were infected with RV, neutrophil infiltration further increased (Figure 2B). In contrast to wild-type mice, RV did not induce eosinophilic inflammation. Finally, we found that, similar to wild type mice, IL-4R KO mice showed OVA-induced airway hyperreactivity which was further exacerbated by RV (Figure 2D and E). (There was no difference in airway reactivity between the wild-type and IL-4R KO OVA/RV groups.) Together, these results show that IL-4 receptor signaling is not required for allergen-induced airway inflammation or hyperresponsiveness. Instead, the immune responses to OVA challenge and RV infection were differentially regulated in the absence of IL-4R signaling, accentuating neutrophilic rather than eosinophilic inflammation.

**Figure 3:**
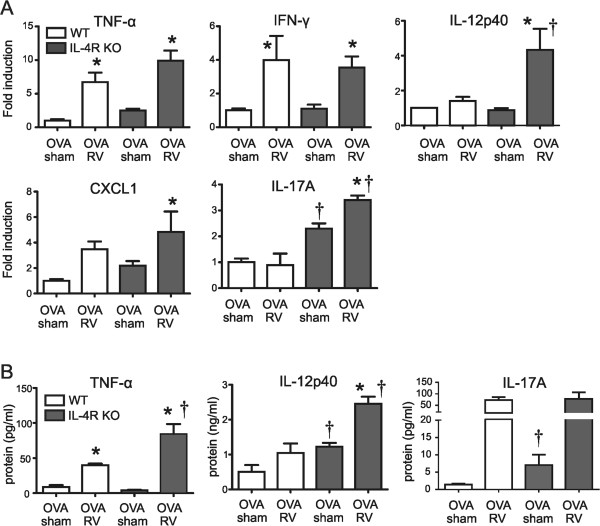
**Cytokine expression in lungs of OVA-treated wild-type and IL-4R KO mice.** Wild-type or IL-4R KO mice were sensitized and challenged with OVA and inoculated with sham or RV. **(A)** After 24 h, lungs were collected and gene expression measured by qPCR. **(B)** TNF-α, IL-12p40 and IL-17A protein in the BAL fluid was assessed with multiplex immune assay. (Mean ± SEM, n = 3-6 each group, *different from sham, *P* < 0.05, one-way ANOVA; †different from wild-type, *P* < 0.05, one-way ANOVA).

To determine the factors driving neutrophilic inflammation in IL-4R KO mice, we analyzed lung mRNA expression by qPCR (Figure 3A). In OVA-treated wild-type mice, RV treatment increased TNF-α and IFN-γ mRNA expression. RV infection of OVA-treated IL-4R KO mice significantly increased TNF-α, IFN-γ, CXCL1, IL-12p40 and IL-17A mRNA levels compared to OVA alone. Finally, compared to OVA-treated, RV-infected wild-type mice, OVA-treated, RV-infected IL-4R KO mice showed increased IL-12p40 and IL-17A mRNA expression. Considering the neutrophil-recruiting ability of TNF-α and CXCL1 [18,19], and the capacity of IL-17A to indirectly stimulate recruitment of neutrophils into the airways via the induction of C-X-C chemokines [10-12,14-16], these results suggest that neutrophilic inflammation in the airways of OVA-treated IL-4R KO mice was mediated, at least in part, by the induction of TNF-α, CXCL1 and IL-17A. We also examined BAL fluid protein levels (Figure 3B). We found that, compared to similarly-treated wild type mice, TNF-α and IL-12p40 levels were significantly higher in the lungs of OVA-treated, RV-infected IL-4R KO mice than wild type mice, consistent with an enhanced type I immune response.

**Figure 4:**
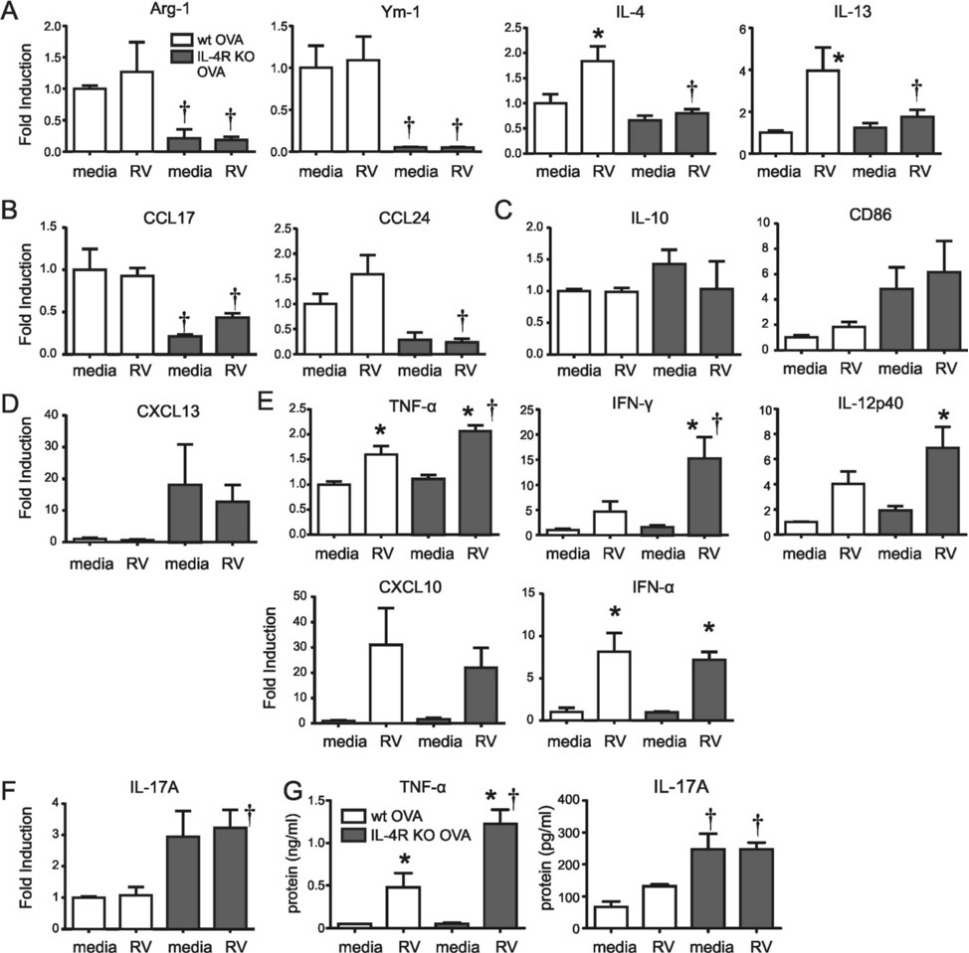
**Differential cytokine expression in RV-stimulated macrophages from OVA-treated wild-type and IL-4R KO mice.** Macrophages were collected from the BAL of OVA-treated wild-type or IL-4RKO mice. Macrophages were selected by allowing adherence to plastic for 2 h. Macrophages were treated with medium or RV (multiplicity of infection, 5) for 1.5 hours. Cells were collected 8 h or 24 h after infection for RNA and protein analysis. Gene expression in macrophage was measured by qPCR. Shown are M2 surface markers and the type 2 cytokines IL-4 and IL-13 **(A)**, the M2a markers CCL17 and CCL24 **(B)**, the M2b markers IL-10 and CD68 **(C)**, the M2c marker CXCL13 **(D)**, the classical M1 markers TNF-α, IFN-γ, IL-12p40 and CXCL10, IFN-α **(E)** and IL-17A **(F)**. **(G)** TNF-α and IL-17A protein levels were assessed with ELISA. (Data represent three independent experiments, mean ± SEM, n = 3-8 each group, *different from sham, *P* < 0.05, one-way ANOVA; †different from wild type, *P* < 0.05, one-way ANOVA).

To examine the specific role of the macrophage in airway neutrophilic responses, we isolated adherent BAL cells (>90% macrophages) from OVA-sensitized and -challenged wild-type and IL-4R KO mice and infected them with RV *ex vivo*. As shown previously [6], macrophages from wild-type mice treated with OVA expressed high levels of the M2 marker Arg 1 and Ym-1, and expressed the type 2 cytokines IL-4 and IL-13 upon RV infection, typical of alternative activation (Figure 4A). In contrast, macrophages from OVA-treated IL-4R KO mice failed to express significant levels of Arg-1 or Ym-1, and did not express IL-4 or IL-13 mRNA with RV infection. IL-4R KO mice also showed reduced expression of the M2a markers CCL17 and CCL24 (Figure 4B). There was no change in the response of IL-10 and CD86, M2b markers (Figure 4C), or CXCL13, an M2c marker (Figure 4D). On the other hand, IL-4R KO macrophages showed more potent “classical” M1 cytokine responses to RV infection, including TNF-α, IFN-γ and IL-12p40 (Figure 4E). Moreover, the basal level of IL-17A gene expression was increased in macrophages from OVA-treated IL-4R KO mice (Figure 4F). Finally, analysis of macrophage supernatants confirmed increased production of TNF-α and IL-17A protein in cells from IL-4R KO mice (Figure 4G). These results suggest that, upon OVA treatment, IL-4R KO macrophages are polarized towards an M1 phenotype, leading to a differential response to RV infection compared to wild-type mice.

**Figure 5:**
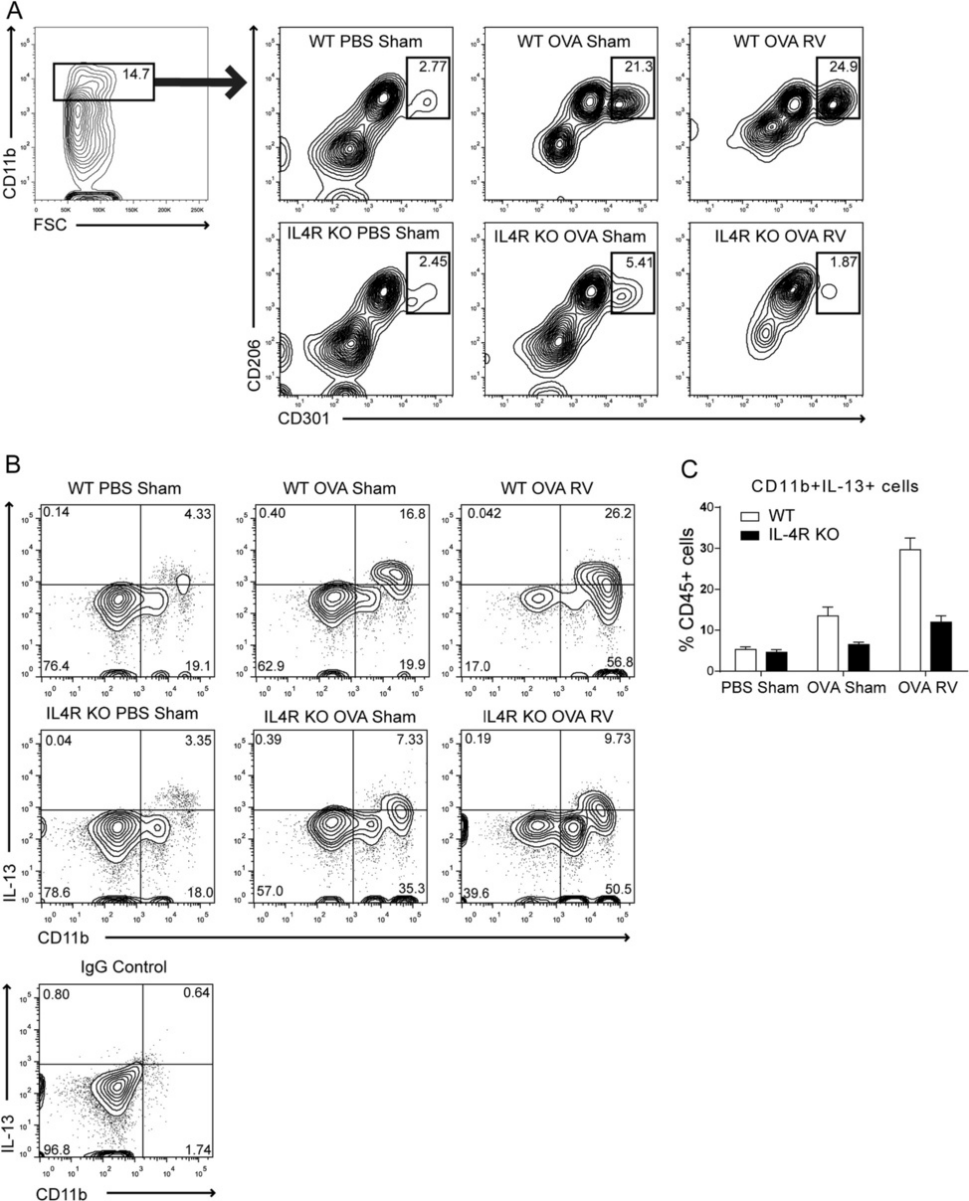
**Differential expansion of CD206+ CD301+ M2-polarized macrophages and IL-13 production in wild-type and IL-4R KO mice.** Eight-week old wild-type or IL-4R KO mice were treated with PBS or OVA by intraperitoneal injection (days 0, 7) and intranasal installation (days 12, 13). Mice were intranasally inoculated with sham or RV on day 14. Lungs were harvested and minced in collagenase IV solution. **(A)** Cells were stained with antibodies against macrophage surface markers and assessed with flow cytometric analysis. CD206- and CD301-double positive cells in the CD11b+ cell fraction are shown. **(B)** Cells were incubated with cell stimulation cocktail for 5 h, stained, and analyzed with flow cytometric method. Expression of CD11b and IL-13 was analyzed among CD45+ cells. The numbers represent the percentage of cells within each quadrant. For the control, IgG was substituted for all antibodies. **(C)** The percentage of CD11b + IL-13+ cells were shown among CD45+ fraction. N = 4, *different from PBS sham group, p < 0.05, ANOVA.

Next, we examined the effect of IL-4R KO on macrophage activation *in vivo*. We reasoned that, in the absence of IL-4R signaling, RV infection of OVA-sensitized and -challenged mice would fail to induce type 2 cytokine production and, instead, increase type 1 and IL-17A cytokine production from conventionally-activated macrophages, augmenting neutrophilic rather than eosinophilic inflammation. Lungs of wild type and IL-4R KO mice were examined by flow cytometric analysis. Lung cell suspensions were stained with antibodies against macrophage surface markers. As we found previously [6], in wild-type mice, there was expansion of a subpopulation of cells expressing the macrophage alternative activation markers CD206 and CD301 after OVA challenge (Figure 5A). Further, IL-13 production by CD11b+ macrophages was increased with OVA treatment and further increased with RV infection (Figure 5B and C). In contrast, in IL-4R KO mice, there was no appearance of a new population of CD206- and CD301-expressing cells (Figure 5A) nor was IL-13 increased after OVA treatment or RV infection (Figure 5B and C). These data suggest that IL-4 signaling is required for alternative activation of lung macrophages and production of effector cytokine IL-13 in this experimental system.

**Figure 6:**
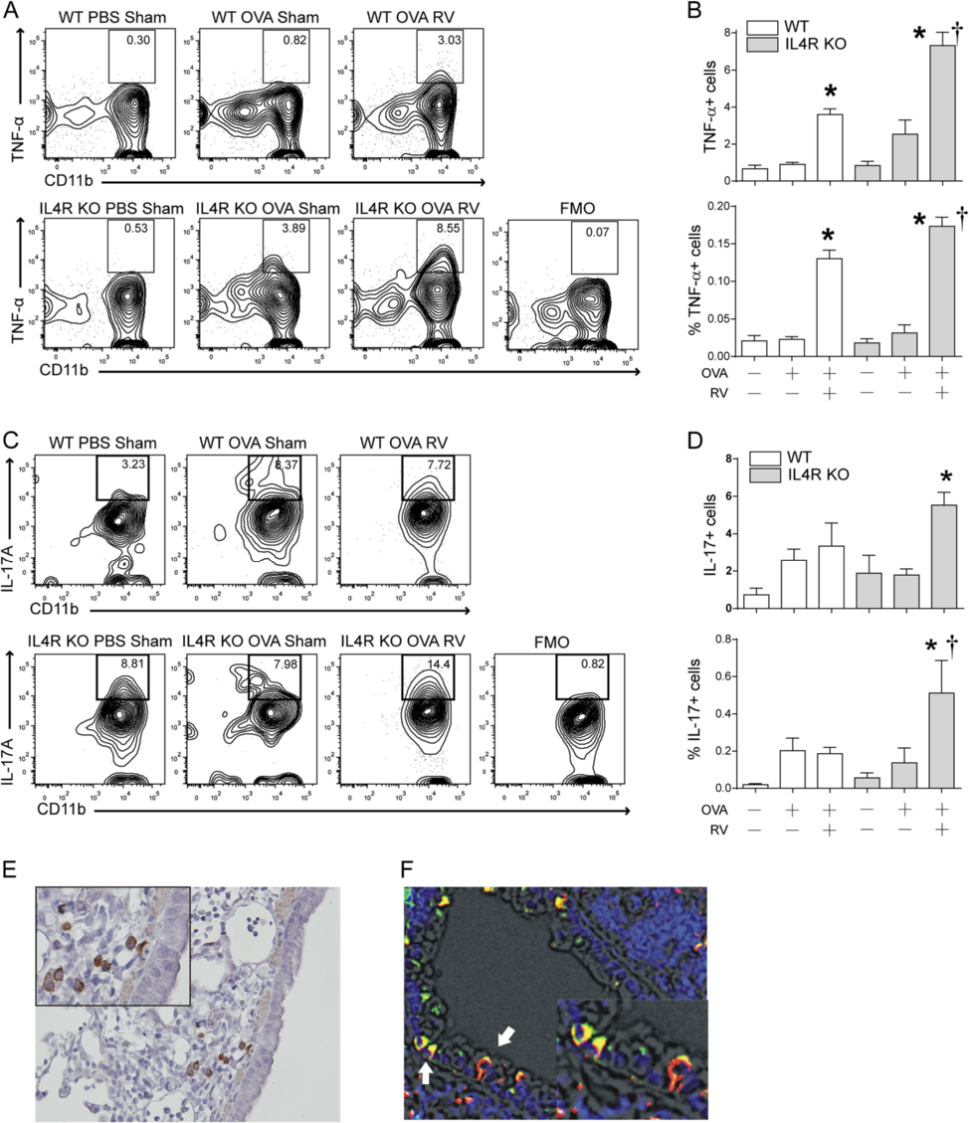
**TNF-α and IL-17A expression in macrophages of wild-type and IL-4R KO mice.** Wild-type or IL-4R KO mice were sensitized and challenged with OVA, and selected mice were inoculated with sham or RV. **(A)** Lungs were harvested and digested with collagenase IV. Cells were stimulated with cell stimulation cocktail for 5 h and stained with antibodies against macrophage surface markers, fixed, permeabilized and incubated with anti-TNF-α. CD11b + TNF-α + cells were analyzed in the CD45+ F4/80+ CD11c- fraction. A fluorescent minus one (FMO) control was utilized to confirm TNF-α signals. In this control, cells were incubated with all antibodies except anti-TNF-α. **(B)** The percentage of CD45+, F4/80+, CD11c-, CD11b+, TNF-α cells in the CD45+ F4/80+ CD11c- fraction (upper panel) and total CD45+ TNF-α + cells (lower panel) were calculated. **(C)** IL-17A producing macrophages were assessed by flow cytometry. Lung cells were stained with anti-IL-17A. CD45+, CD68+, F4/80+, CD11c- cells were analyzed for CD11b and IL-17A. An FMO control (all antibodies except anti-IL-17) was used to confirm IL-17 signals. **(D)** The percentage of CD45+, CD68+, F4/80+, CD11c-, CD11b+, IL-17A + cells in the CD45+ CD68+ fraction (upper panel) and total CD45+ IL-17A + cells (lower panel) were calculated. **(E)** Lung sections were stained with anti-IL-17A antibody. Immunohistochemistry shows DAB staining of round cells in the airway subepithelium. **(F)** Lungs were stained with AF555-conjugated anti-CD68 (red) and AF488-conjugated anti-IL-17A (green). Nuclei were stained with DAPI (blue). Immunofluorescence shows colocalization (yellow), indicating IL-17A production by CD68+ macrophages. (Mean ± SEM, n = 3-5 each group, *different from sham, *P* < 0.05, one-way ANOVA; †different from wild-type, *P* < 0.05, one-way ANOVA).

We asked whether type 1 cytokine production is increased in macrophages from OVA-treated IL-4R KO mice *in vivo*. We collected the lungs of wild type and IL-4R KO mice, stained for TNF-α and analyzed with flow cytometry. We sorted for CD11c- CD11b+ non-resident, exudative macrophages [20,21]. We found that, compared to wild-type mice, TNF-α producing CD45+, F4/80+, CD11c-, CD11b+ macrophages were increased in OVA-treated IL-4R KO mice, and these cells were further increased with RV infection (Figure 6A and B).

To test whether macrophages secrete IL-17A *in vivo*, we examined the lungs of wild type and IL-4R KO mice by flow cytometric analysis. We found that CD45+, TCRβ-, CD68+, F4/80+, CD11c-, CD11b+, IL-17A+ macrophages were increased in naïve IL-4R KO mice compared to wild-type mice, and further enhanced with RV infection (Figure 6C and D). We also employed immunohistochemistry and immunofluorescence microscopy to confirm IL-17A localization in lungs from OVA-treated IL-4R KO mice. IL-17A+ monocytic cells were evident in the subepithelium (Figure 6E). IL-17A co-localized with CD68, a macrophage marker (Figure 6F).

## Discussion

Viral-induced exacerbations are a major cause of morbidity in asthma. RVs comprise approximately 50% of the viruses isolated. However, the precise mechanisms underlying RV-induced asthma exacerbations are not known. RV, unlike influenza and other viruses, causes minimal if any cytotoxicity [22,23]. The current explanation is that RV infection induces the release of chemokines from airway epithelial cells, thereby attracting inflammatory cells to the airways. However, it is also conceivable that RV directly infects airway inflammatory cells. Several studies have examined the interaction of monocytic cells and RV *in vitro*[24-32]. Recently, we found that, in both mice and humans, RV colocalizes with monocytes *in vivo*[6,33]. Following infection of OVA-sensitized and -challenged mice, we found that RV colocalized with eotaxin-producing, CD68+ lung macrophages*.* Compared to cells from untreated mice, BAL macrophages from allergen-treated mice showed increased expression of type 2 and decreased expression of type I cytokines in response to ex vivo RV infection, indicating a shift from M1 to M2 activation status. Finally, depletion of macrophages using clodronate liposomes reduced RV-induced eosinophilic inflammation and airway hyperreactivity, suggesting that RV causes asthma exacerbations in part by infection of alternatively-activated macrophages.

**Figure 7:**
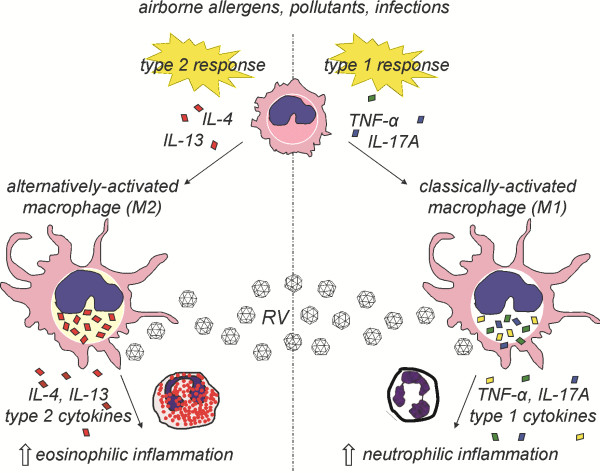
**Macrophage activation state determines the response to subsequent rhinovirus infection.** Upon airway injury, individuals with a type 2-predominant immune response experience eosinophilic airway inflammation which is heightened by RV infection. Alternatively, individuals with a type 1/IL-17-predominant response (analogous to IL-4R KO mice) demonstrate neutrophilic inflammation which is exacerbated by RV infection.

We hypothesized that IL-4 signaling drives the state of macrophage activation and determines the pattern of RV-induced exacerbation in mice with allergic airways disease. To test this in an animal model, we examined the effect of RV infection on OVA-sensitized and -challenged wild-type and IL-4R KO mice. We anticipated that, in the absence of IL-4/IL-13 signaling, RV infection would increase type 1 cytokine production from conventionally-activated macrophages, augmenting neutrophilic rather than eosinophilic inflammation. We found that OVA increased the number of CD206+, CD301+ M2-polarized lung macrophages and IL-13+ CD11b+ cells in wild type but not IL-4R KO mice. These cells produced In addition, unlike OVA-exposed wild-type mice with mixed neutrophilic and eosinophilic inflammation, OVA treatment of IL-4R KO mice induced airway inflammation which was almost exclusively neutrophilic in character. When OVA-treated IL-4R KO mice were infected with RV, neutrophil infiltration further increased. Neutrophilic inflammation was associated with increased lung type 1 cytokine expression, and BAL macrophages expressed type 1 cytokines in response to RV infection *ex vivo.* Together, these results show that IL-4 signaling is required for lung macrophage M2 polarization, and that macrophage polarization state determines the response to RV infection (Figure 7).

Many studies have demonstrated the importance of IL-4R signaling in the regulation of the immune response, including the response of lung macrophages [34-36]. However, fewer studies have examined the role of M2-polarized alternatively-activated macrophages in the response to pulmonary infection. The loss of IL-4R-, STAT1-dependent alternative activation has been shown to confer resistance to RSV-induced lung injury [37], pulmonary cryptococcosis [38-40], *Mycobacterium tuberculosis*[41] and severe acute respiratory syndrome coronavirus (SARS-CoV) [42]. Resistance has been attributed to the higher potential of classically activated macrophages to produce nitric oxide [40]. In contrast, we showed that, in allergen-sensitized and -challenged mice, either classically- and alternatively-active macrophages may contribute to RV-induced airway inflammation and hyperresponsiveness. We speculate that M1 macrophages are not protective against RV because the inflammatory response does not depend on viral load [43].

In this study, we found that IL-17A was increased in the lungs of OVA-treated IL-4R KO mice and expressed by BAL and lung macrophages infected with RV*.* IL-17A plays an important role in the recruitment and activation of neutrophils following bacterial infection [11]. IL-17A indirectly stimulates recruitment of neutrophils into the airways via the induction of C-X-C chemokines [10-12,14-16]. Production of IL-17A was first reported in CD4+ cells [44]. IL-17A-producing Th17 cells are regarded as a distinct subset of T cells, divergent from Th1 and Th2 cells. It is now established that neutrophils, eosinophils and macrophages also produce IL-17A [14,45,46]. It was recently shown that IL-13 signaling inhibits IL-17A production from CD4+ Th17 cells [47]. Thus, it is likely that, in our study, IL-17A production was derepressed in IL-4R KO mice deficient in IL-13 signaling. Further, we found that, in the absence of IL-4R signaling, BAL macrophages from OVA-treated mice were polarized to produce IL-17A as well as type 1 cytokines. Macrophage IL-17A production likely contributed to the observed neutrophilic inflammation.

We found that OVA-treated IL-4R KO mice showed neutrophilic airway inflammation, essentially constituting a model of allergic, type 1 cytokine-driven neutrophilic asthma. IL-4 KO mice have previously shown increased neutrophils upon ovalbumin exposure [35]. Although the allergic type 2 immune response has been considered as a hallmark of asthma, only 50% of asthma cases are due to eosinophilic inflammation, the rest showing airway neutrophils [48]. This type of asthma has been associated with specific asthma phenotypes including severe asthma, corticosteroid-resistant asthma, nocturnal asthma and occupational asthma [49-57]. Although non-allergic stimuli, for example, lipopolysaccharide and ozone [58-60], have been associated with neutrophilic airway inflammation, our data are consistent with the notion that a subset of allergic patients may experience neutrophilic rather than eosinophilic airway inflammation due to the influence of type 1 cytokines and IL-17A. While we did not determine the precise mediators driving airway hyperresponsiveness in the absence of IL-4R signaling, we suspect that macrophage- and neutrophil-derived TNF-α plays a role. TNF-α mRNA and protein expression were increased in the lungs and lung macrophage supernatants of OVA-exposed, RV-infected IL-4R KO mice. In addition, we have shown that TNF-α is required for RV-induced airways responsiveness in naïve mice [61].

We would like to note some limitations of our animal model. Species differences between human and mouse partially restrict viral replication, thereby requiring a higher inoculum However, we have demonstrated viral replication and IFN responses following inoculation of mice with human RV [17]. We have also shown that the inflammatory response to RV infection is greater than that to UV-irradiated virus, sham-infected HeLa cell lysate, and filtrates from RV-infected HeLa cell lysates [62]. Another potential concern relates to our model’s dependence on minor group viruses. However, effects of RV1B infection on wild-type mice are indistinguishable from those of RV16, a major group virus, on transgenic human ICAM-1 mice [63]. Major and minor group viruses induce nearly identical patterns of gene expression in cultured airway epithelial cells [64]. Analysis of all RV genomes revealed that RV1 and 16 are highly homologous and respond similarly to antiviral compounds [65], implying that the distinction between some major and minor group strains may not be clinically relevant.

## Conclusion

We showed that, in contrast to sensitized and challenged wild-type mice with mixed neutrophilic and eosinophilic inflammation, IL-4R KO mice sensitized and challenged with OVA have significant neutrophilic inflammation which is further enhanced by RV infection. Macrophages from OVA-treated IL-4R KO mice showed an M1 polarization pattern and expressed type 1 cytokines and IL-17A in response to RV infection. We conclude that, in allergen-sensitized mice, the macrophage activation state determines the response to RV infection.

## Abbreviations

OVA: Ovalbumin; RV: Rhinovirus; M1 macrophage: Classically-activated macrophage; M2 macrophage: Alternatively-activated macrophage; IL-4R KO: IL-4 receptor knockout; CD: Cluster of differentiation; IL: Interleukin; IFN: Interferon; CXCL: CXC chemokine ligand; CCL: CC chemokine ligand; TNF: Tumor necrosis factor; Arg-1: Arginase-1; TCR: T cell receptor; DAPI: 4′,6-diamidino-2-phenylindole.

## Competing interests

Dr. Hershenson has performed consulting work for Boehringer-Ingelheim and Almirall. Dr. Sajjan has received research funding from Quercegen Pharmaceuticals. These entities had no role in the preparation of this manuscript.

## Authors’ contributions

JYH, JKB, US, and MBH designed research. JYH, YC, JS, QC, JL, ATC, AMG and JKB conducted experiments. JKB, US, and MBH provided help with data interpretation. JYH and MBH wrote the paper. All authors read and approved the final manuscript.
